# Characterization of Antimicrobial Resistance and Potential Zoonotic Risk in Uropathogenic *Escherichia coli* Isolated from Companion Animals, with Genomic Analysis of Virulence Determinants in a Representative Isolate

**DOI:** 10.3390/tropicalmed11040101

**Published:** 2026-04-13

**Authors:** Asanka R. DeZoysa, Madeline Kwan, Lekshmi K. Edison, Rebecca Barber, Lisa Glick, Thomas Denagamage, Subhashinie Kariyawasam

**Affiliations:** 1 Department of Large Animal Clinical Sciences, College of Veterinary Medicine, University of Florida, Gainesville, FL 32610, USA; adezoysa@ufl.edu (A.R.D.); tdenagamage@ufl.edu (T.D.); 2 Department of Comparative, Diagnostic and Population Medicine, College of Veterinary Medicine, University of Florida, Gainesville, FL 32610, USA; m.kwan@ufl.edu (M.K.); edison.le@ufl.edu (L.K.E.); 3 Clinical Microbiology/Parasitology/Serology Laboratory, University of Florida-Veterinary Hospitals, College of Veterinary Medicine, University of Florida, Gainesville, FL 32610, USA; rbarber1@ufl.edu (R.B.); lisa.glick@ufl.edu (L.G.)

**Keywords:** antimicrobial resistance, biofilm formation, companion animals, urinary tract infection, uropathogenic *Escherichia coli*, virulence genes

## Abstract

Uropathogenic *Escherichia coli* (UPEC) is a leading cause of urinary tract infections (UTIs) in companion animals. This study characterized 42 UPEC isolates recovered from dogs and cats at the University of Florida, College of Veterinary Medicine Diagnostic Laboratories between 2023 and 2024, focusing on antimicrobial resistance (AMR), virulence gene profiles, biofilm-forming ability, and phylogroup distribution of the isolates. Antimicrobial susceptibility testing (AST) showed that 40.48% of the isolates were resistant to at least one of the tested antibiotics, and 9.52% exhibited multidrug resistance (MDR). Phylogroup B2 was predominant (69.05%), and 61.90% of isolates demonstrated strong biofilm formation in artificial human urine. Virulence gene analysis revealed the presence of genes mediating adhesion (*fim*, *pap*, *sfa*), iron acquisition (*fyuA*, *iro*), biofilm formation (*csg*, *bcs*, *pga*, *ycg/ymg*), motility (*fli*, *mot*, *flh*), and stress response (*oxyR*, *soxR/S*, *kat*). Multiple plasmids carrying AMR and virulence determinants were also identified. The co-occurrence of the traits underscores the potential for persistent and recurrent infections, which can complicate therapeutic outcomes and facilitate horizontal gene transfer (HGT). The detection of antimicrobial-resistant, highly virulent UPEC strains possessing human UPEC traits in companion animals suggests the risk of zoonotic and reverse-zoonotic transmission, particularly in households with close pet–owner interactions. These findings emphasize the importance of judicious antimicrobial use, routine molecular surveillance, and integrated One Health strategies to mitigate the veterinary and public health threats associated with UPEC infections in companion animals.

## 1. Introduction

Urinary tract infections (UTIs) are a common clinical diagnosis in companion animal practice in the United States and worldwide [1]. The estimated lifetime incidence of UTIs is approximately 14% in dogs and 3% to 19% in cats [2]. UTIs occur as a consequence of microbial colonization, adherence to, and proliferation within the urinary tract. The most common clinical manifestations of UTIs include acute cystitis and pyelonephritis [3], whereas asymptomatic bacteriuria is a frequently encountered diagnostic condition. UTIs are also highly prevalent in humans and represent one of the most common infectious diseases in the United States, accounting for more than 10.5 million healthcare visits annually [4]. Epidemiological data further indicate that nearly one in two women will experience at least one episode of UTI during their lifetime [5].

UTIs are primarily associated with bacterial pathogens such as *Escherichia coli*, *Klebsiella pneumoniae*, *Proteus mirabilis*, *Enterococcus faecalis*, and *Staphylococcus saprophyticus*. Among these, *E. coli* C represents the predominant etiological agent of UTIs [6]. Companion animals acquire uropathogenic *E. coli* (UPEC) through endogenous colonization, as these strains are frequently present in the intestinal microbiota and may ascend from the perineal region to the urinary tract. Additional sources of acquisition include environmental reservoirs such as contaminated kennels, litter boxes, and water, as well as iatrogenic introduction during urinary catheterization or surgical procedures. Host-related predisposing factors, including diabetes mellitus, urolithiasis, chronic kidney disease, and immunosuppression, increase the likelihood of infection. Importantly, molecular epidemiological studies have demonstrated clonal similarities between UPEC isolates from companion animals and their owners, suggesting that zoonotic and reverse-zoonotic transmission may contribute to the circulation of UPEC in shared environments [5].

Effective management of UTIs in companion animals requires a proper diagnosis and rational selection of antimicrobial therapy. Antimicrobial treatment often includes beta-lactam antibiotics, trimethoprim-sulfonamide combinations, aminoglycosides, and fluoroquinolones. However, the efficacy of these agents can vary based on antimicrobial resistance (AMR) patterns, as many UPEC isolates from companion animal UTIs show multidrug resistance (MDR) [7]. Therefore, urine culture and antimicrobial susceptibility testing (AST) are essential for guiding appropriate therapy, preventing treatment failures, and reducing the emergence of further resistance. This is particularly important due to AMR represents a major global public health challenge, largely driven by inappropriate or excessive antimicrobial use.

The majority of UPEC isolates harbor virulence genes that enhance pathogenicity, facilitate evasion of host immune defenses, enable survival under adverse host conditions, and promote biofilm formation, thereby supporting bacterial persistence within the urinary tract and contributing to severe clinical disease [8]. Detection of these genes can aid in predicting strain pathogenicity and guiding the development of targeted therapies. Therefore, this study aimed to characterize UPEC isolates from dogs and cats diagnosed with UTI by (i) determining their phenotypic AMR patterns, (ii) identifying virulence genes associated with pathogenicity, and (iii) assessing their ability to form biofilms in artificial human urine to evaluate the potential zoonotic risk of these isolates.

## 2. Materials and Methods

### 2.1. Sample Collection

A total of 42 UPEC isolates were obtained from glycerol stocks maintained in the Clinical Microbiology, Parasitology, and Serology Laboratory at the University of Florida-Veterinary Hospitals (UF-VH). These isolates originated from canine and feline patients admitted to the Small Animal Hospital with a clinical diagnosis of UTI. Urine samples from these patients had been submitted to the laboratory for culture confirmation and AST testing between January 2023 and December 2024. Demographic information, including species, age, and sex, was recorded.

### 2.2. Antimicrobial Susceptibility Testing (AST)

AST of isolates was performed using the Kirby-Bauer disk diffusion method. Commercial antibiotic disks (Oxoid™, Thermo Fisher Scientific, Waltham, MA, USA), including cefoxitin (FOX30), cefepime (FEP30), tetracycline (TE30), doxycycline (D30), gentamicin (GM10), amikacin (AN30), imipenem (IPM10), and chloramphenicol (C30), were selected based on their clinical relevance for the treatment of UTIs in companion animals. *Escherichia coli* ATCC 25922 (American Type Culture Collection, Manassas, VA, USA) was used as the quality control strain [9] for AST. Zone diameter values were interpreted according to the Clinical and Laboratory Standards Institute (CLSI) guidelines [10]. The results were recorded in Microsoft Excel for analysis. Isolates were classified as multidrug-resistant (MDR) if they exhibited resistance to at least one antimicrobial agent in three or more antimicrobial classes [11].

### 2.3. Multiplex PCR for Urovirulence Genes

Genomic DNA was extracted from the isolates using the InstaGene Matrix kit (Bio-Rad Laboratories, Hercules, CA, USA) according to the manufacturer’s instructions. A previously established multiplex PCR protocol [12] was used to detect the urovirulence genes *vat*, *fyuA*, *chuA*, and *yfcV* in the 42 isolates included in the study. Four primer sets (Supplementary File S1) targeting these genes, which have been previously used to assess the urovirulence potential of human UPEC, were combined in a single reaction for multiplex PCR using the QIAGEN^®^ Multiplex PCR Kit (Qiagen, Germantown, MD, USA). Each 25 µL PCR reaction mixture contained 12.5 µL of 2× QIAGEN Multiplex PCR Master Mix, 0.1 µM each of forward and reverse primers for the four target genes, and 20 ng of template DNA. UPEC strains CFT073 (NCBI accession # CP051263) and UTI89 (NCBI accession # NC007946) were used as positive controls, and PCR-grade water served as the negative control. Thermocycling was performed with an initial denaturation at 95 °C for 15 min, followed by 30 cycles of denaturation at 94 °C for 30 s, annealing at 63 °C for 1.5 min, and extension at 72 °C for 1.5 min with a final extension at 72 °C 10 min. Following PCR amplification, 10 µL of each amplicon, along with the positive and negative controls, were subjected to electrophoresis on a 2% (*w*/*v*) agarose gel.

### 2.4. Quadruplex PCR for Phylo-Typing

A previously published quadruplex PCR protocol [13] was used to determine the phylogenetic groups of the 42 isolates. Four primer pairs (Supplementary File S1) targeting the genes *chuA*, *yjaA*, and *arpA*, and the DNA region TspE4.C2 were used in a single multiplex reaction with the QIAGEN^®^ Multiplex PCR Kit (Qiagen). Each 25 µL PCR mixture contained 12.5 µL of 2× QIAGEN Multiplex PCR Master Mix, 0.1 µM each of forward and reverse primers for the *chuA*, *yjaA*, TspE4.C2, 0.2 µM of *arpA* primer set and 20 ng of template DNA. The UPEC strains CFT073 and UTI89 were used as positive controls, while PCR-grade water was used as a negative control. Thermocycling conditions consisted of an initial denaturation at 95 °C for 15 min, followed by 30 cycles of denaturation at 94 °C for 30 s and annealing at 63 °C for 1.5 min and extension at 72 °C for 1.5 min, with a final extension at 72 °C for 10 min. Following PCR amplification, 10 µL of each PCR product, along with the positive and negative controls, were analyzed by electrophoresis on a 2% (*w*/*v*) agarose gel.

### 2.5. Biofilm Formation Assay

Biofilm formation was assessed using the tube-adherence method, as previously described [14]. Briefly, 1 mL of Luria–Bertani (LB) broth was inoculated with 2–3 colonies of each UPEC isolate and incubated overnight at 37 °C in a shaking incubator at 200 rpm. *E. coli* CFT073 and UTI89 strains were used as positive controls. A 10 µL volume of each overnight UPEC culture was inoculated into 1 mL of commercially available sterile pooled human urine (Innovative Research, Novi, MI, USA) in sterile glass tubes. Each assay was performed in triplicate to ensure the reproducibility of the results. Uninoculated artificial urine was used as a negative control. All inoculated tubes, along with the positive and negative controls, were incubated statically at 37 °C for 24 h. After incubation, planktonic growth was visually assessed and recorded for further analysis. The supernatant was gently decanted without disturbing the adhered biofilms. Each tube was washed with 1 mL of sterile Phosphate-Buffered Saline (PBS, Thermo Fisher Scientific, Waltham, MA, USA) by gentle shaking for 30 s to remove non-adherent bacteria. The wash solution was discarded, and the tubes were air-dried for 2 min. Subsequently, 1 mL of 0.1% Crystal Violet (CV—Certified Biological Stain; Thermo Fisher Scientific) solution was added to the tubes and incubated at room temperature for 35 min. The CV solution was then removed, and the tubes were rinsed with 1 mL of PBS and inverted on a paper towel to dry for 15 min. The presence of a visible film lining the bottom and walls of the glass tubes was considered indicative of biofilm formation.

Quantification of biofilm production was performed as the final step of the assay. A 1 mL of 95% ethanol was added to each tube to solubilize the bound CV. From each tube, 200 µL of the ethanol-dissolved stain was transferred to a 96-well plate, along with positive and negative controls. Optical density (OD) at 595 nm (OD_595_) was measured using a spectrophotometer. The cut-off OD (ODc) was calculated using the formula described by Harika et al. (2020) [15] as follows:

ODc=Average OD of negative control + 3 × SD of negative control

where *SD* represents the standard deviation

### 2.6. Whole-Genome Sequencing and Comparative Analysis with Human UPEC Reference Strains

For a comprehensive analysis of AMR genes and virulence-associated genes, one representative UPEC isolate, UPEC957C (Sample #S7), was selected from the 42 total isolates. The selection criteria were as follows: (i) phenotypic resistance to more than one antimicrobial classes; (ii) presence of all urovirulence genes (*vat*, *fyuA*, *chuA*, and *yfcV*) as determined by multiplex PCR; (iii) assignment to phylogenetic group B2 based on *E. coli* phylotyping PCR; and (iv) strong biofilm-forming ability.

Pure culture of canine UPEC strain *E. coli* UPEC957C was submitted to SeqCenter (Pittsburgh, PA, USA) for whole-genome sequencing using a hybrid approach that combined Illumina Sequencing for short-read data and Oxford Nanopore Technology (ONT) for long-read data generation. Illumina libraries were prepared using the tagmentation-based Illumina DNA Prep kit (Illumina, Inc., San Diego, CA, USA) with custom 10-bp UDIs (target insert ~280 bp) without additional fragmentation or size selection and sequenced on a NovaSeq X Plus sequencer (Illumina). Demultiplexing, quality control, and adapter trimming were performed using bcl-convert v.4.2.4. ONT long-read libraries were prepared PCR-free using the SQK-NBD114.96 ligation kit (Oxford Nanopore Technologies, Oxford, UK) and NEBNext^®^ Companion Module (New England Biolabs, Inc., Ipswich, MA, USA), sequenced on GridION/PromethION sequencer using R10.4.1 flow cells (Oxford Nanopore Technologies) in 400 bps mode, and basecalled with Dorado v0.5.3 (sup and 5 mC/5 hmC models) with subsequent demultiplexing and FASTQ extraction using dorado demultiplex and samtools fastq (v.1.17). Residual adapters were removed using Porechop v.0.2.4, and sequencing metrics were generated using Fasttp (v0.23.4) Hybrid de novo assemblies were generated with Flye v.2.9.2 (nano-hq model; genome size 6 Mb; 50× longest-read coverage), polished with Illumina reads using Pilon v.1.24, and low-coverage (≤15×) long-read contigs were removed. Assemblies were assessed for circularization using Circulator v.1.5.5, annotated with Bakta v.1.8.1, and evaluated using QUAST v.5.2.0, producing the final FASTA, GFF, and GenBank files.

Upon receiving the sequence data, whole-genome assembly and annotation were performed using CLC Genomics Workbench v.25.0.1 (Qiagen). The assembled genome was analyzed to identify AMR genes, virulence factors, and plasmid replicons using the ResFinder, CARD, VirulenceFinder, Virulence Factor Database (VFDB), and PlasmidFinder databases. Multilocus sequence typing (MLST) was conducted using the PubMLST server (https://pubmlst.org/bigsdb?db=pubmlst_rmlst_seqdef_kiosk, accessed on 20 August 2025), and phylogenetic relationships based on single-nucleotide polymorphism (SNP) were inferred using CSI Phylogeny 1.4 (https://cge.food.dtu.dk/services/CSIPhylogeny/, accessed on 26 August 2025) with default settings and FigTree graphical viewer (https://tree.bio.ed.ac.uk/software/figtree/, accessed on 26 August 2025), employing the selected canine UPEC isolate *E. coli* UPEC957C (Sample #S7) as the reference genome and UTI89 and CFT073 as comparator genomes.

The whole-genome shotgun reads of *E. coli* UPEC957C have been deposited at the National Center for Biotechnology Information (NCBI) GenBank under the BioProject PRJNA1417591 with accession number JBUECA000000000.

## 3. Results

### 3.1. Demographic Characteristics of Selected Samples

Among the 42 UPEC isolates, the majority originated from canines (*n* = 34, 80.95%), while feline UPEC were accounted for 19.05% (*n* = 8). Regarding sex distribution, female and male animals represented 66.7% (*n* = 28) and 33.3% (*n* = 14), respectively. The ages of the dogs ranged from 1 month to 17 years, whereas cats ranged from 2 to 20 years; age information was unavailable for one canine and one feline patient. Among the canine isolates, 9 (26.5%) were from animals <1 year of age, 12 (35.3%) were from animals aged 1–9 years, and 12 (35.3%) were from animals ≥10 years. Among the feline isolates, 4 (50.0%) were from animals aged 1–9 years old and 3 (37.5%) from animals ≥10 years. The demographic characteristics of the host species from which the UPEC isolates were obtained are summarized in Table 1. Detailed demographic information for patients from whom the urine samples were derived is included in Supplementary File S2.

### 3.2. Phenotypic Antimicrobial Resistance Patterns of Isolates

**Figure 1:**
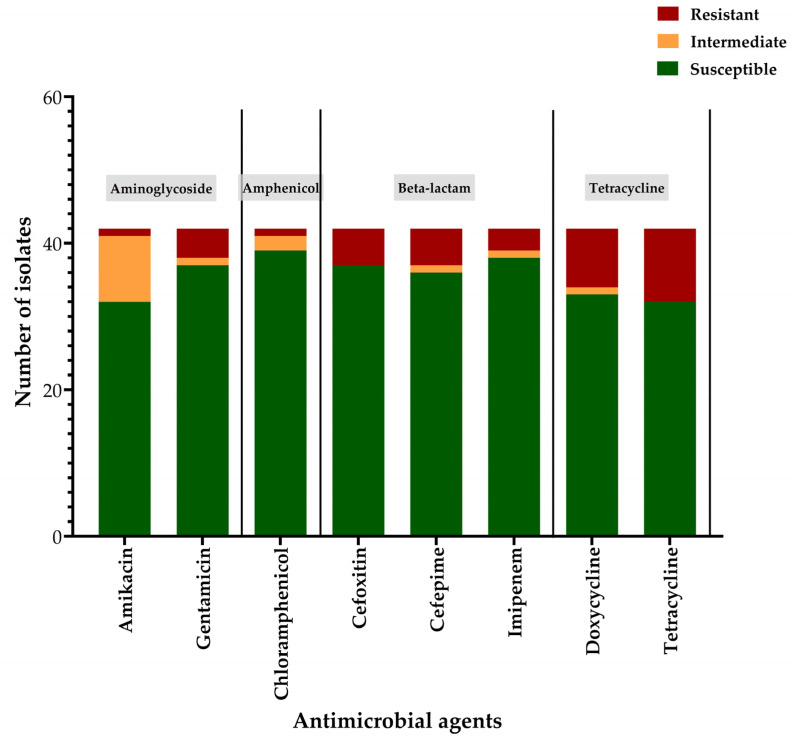
Phenotypic antimicrobial susceptibility profiles of uropathogenic *Escherichia coli* (UPEC) isolates. The bar chart illustrates the phenotypic resistance patterns of 42 UPEC isolates tested against eight antimicrobial agents representing four major antibiotic classes, aminoglycosides (amikacin, gentamicin), amphenicols (chloramphenicol), beta-lactams (cefoxitin, cefepime, imipenem), and tetracyclines (doxycycline, tetracycline) using the Kirby-Bauer disk diffusion method. Each bar represents the number of isolates classified as resistant (red), intermediate (orange), or susceptible (green) according to CLSI interpretive criteria.

Among the 42 isolates, 17 (40.48%) exhibited resistance to at least one antibiotic, while MDR was observed in 9.52% (4/42) of the isolates. Resistance to individual antibiotics was as follows: amikacin (AN30), 2.38% (1/42); gentamicin (GM10), 9.52% (4/42); chloramphenicol (C30), 2.38% (1/42); cefoxitin (FOX30), 11.90% (5/42); cefepime (FEP30), 11.90% (5/42); imipenem (IPM10), 7.14% (3/42); doxycycline (D30), 19.05% (8/42) and tetracycline (TE30), 23.81% (10/42). Figure 1 illustrates the phenotypic resistance profiles of the 42 UPEC isolates to eight antimicrobial agents. Detailed results, including all the raw data, are provided in Supplementary File S3.

### 3.3. Distribution of Urovirulence Genes Implicated in Human UTI

**Figure 2:**
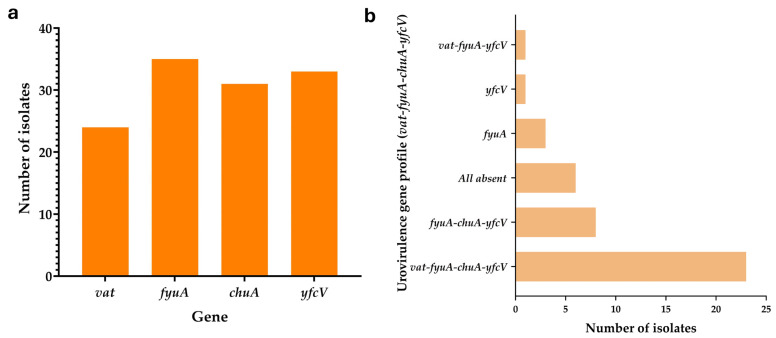
Distribution of urovirulence genes among uropathogenic *Escherichia coli* (UPEC) isolates. (**a**) The bar graph represents the frequency of four key urovirulence genes, *vat*, *fyuA*, *chuA*, and *yfcV*, detected among 42 UPEC isolates. (**b**) The horizontal bar graph shows the distribution of six different combinations of these urovirulence genes among the isolates.

Among the 42 canine and feline clinical UPEC isolates examined, 23 (54.76%) possessed all four human urovirulence genes (*vat*, *fyuA*, *chuA*, and *yfcV*) detected by multiplex PCR. Eight isolates (19.05%) carried *fyuA*, *chuA*, and *yfcV*, but lacked *vat*. One isolate harbored *vat*, *fyuA*, and *yfcV* while lacking *chuA*. Three isolates carried only the *fyuA* gene, one isolate carried only *yfcV*, and six isolates (14.29%) lacked all four genes. The distribution of four urovirulence genes is shown in Figure 2. The gene presence–absence matrix for all 42 isolates, along with the corresponding PCR gel documentation for all 42 samples, are provided in Supplementary Files S4 and S5, respectively.

### 3.4. Phylogroup Classification and Distribution Among Clinical Isolates

**Figure 3:**
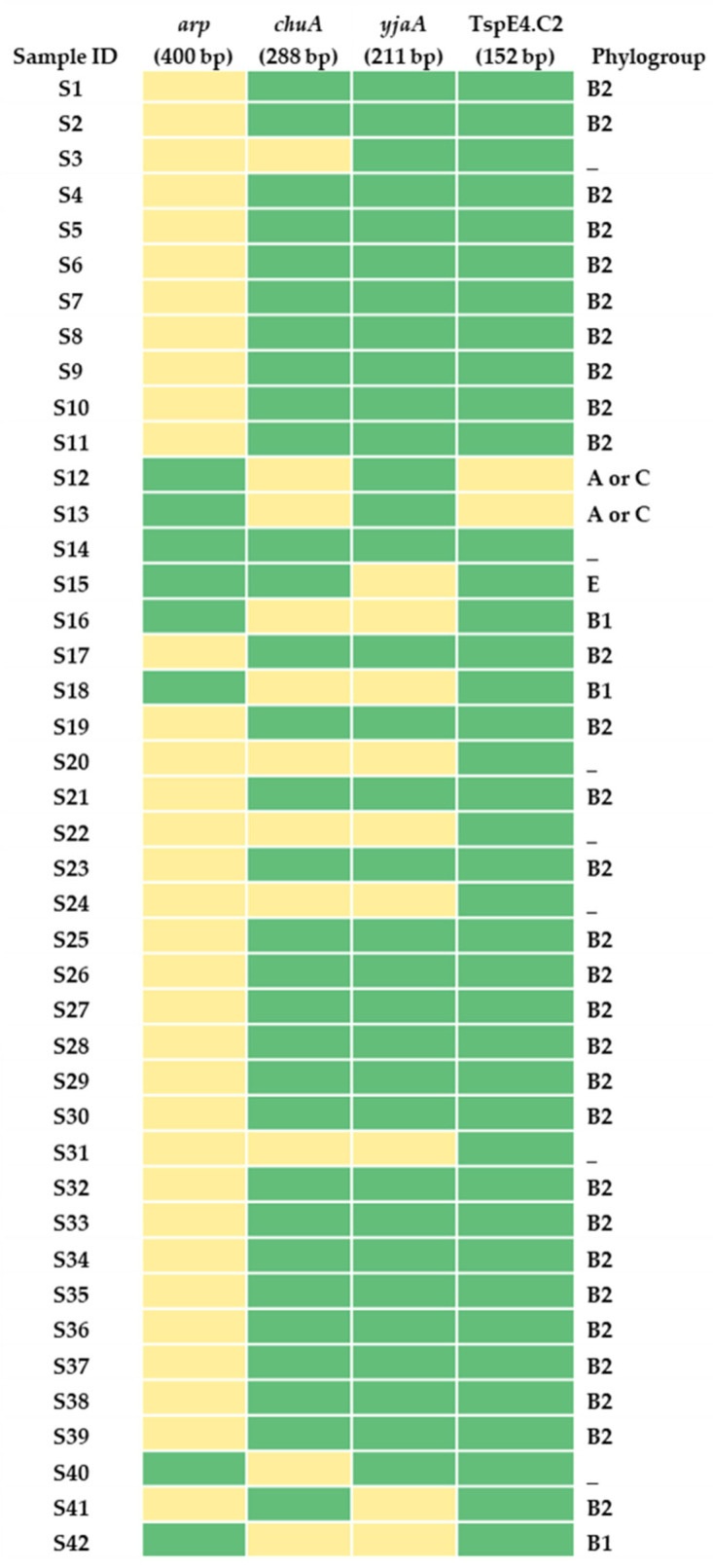
Distribution of phylogenetic group assignment among uropathogenic *Escherichia coli* (UPEC) isolates. The heat map depicts the presence (green) and absence (yellow) of four phylogenetic markers, *arp*, *chuA*, *yjaA*, and TspE4.C2 across 42 UPEC isolates. Each row represents an individual isolate, while columns correspond to the phylogenetic markers analyzed. The rightmost column indicates the phylogenetic group assignment for each isolate based on the Clermont phylo-typing scheme.

Based on the quadruplex PCR method described previously [13], 29 of the 42 UPEC isolates (69.05%) were assigned to phylogroup B2, 3 isolates (7.14%) to phylogroup B1, 2 isolates (4.76%) to phylogroups A or C, and 1 isolate (2.38%) to phylogroup E. The remaining 7 isolates (16.67%) could not be assigned to any of the defined phylogroups according to the Clermont classification scheme. The distribution of the four genetic markers (*arpA*, *chuA*, *yjaA*, and TspE4.C2) used for phylogroup determination, along with the corresponding phylogroup assignments for the 42 UPEC isolates, is presented in Figure 3. The detailed gene composition data used for phylogroup assignments are provided in Supplementary File S6. The corresponding phylogrouping PCR gel images are included in Supplementary File S7.

### 3.5. Biofilm Formation Patterns of Clinical UPEC Isolates

**Figure 4:**
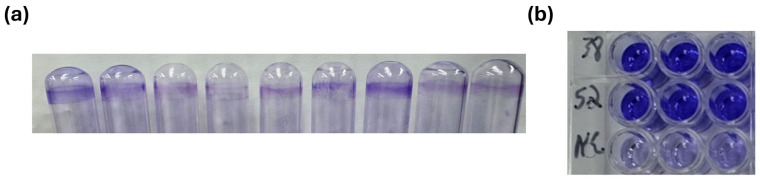
Qualitative (**a**) and quantitative (**b**) assessments of biofilm formation by uropathogenic *Escherichia coli* (UPEC) isolates using crystal violet staining. (**a**) Representative image showing biofilms formed by UPEC isolates on the inner surface of glass tubes following crystal violet staining. The intensity of the violet ring indicates the degree of biofilm formation on the glass surface. (**b**) Crystal violet–stained biofilms formed in 96-well microtiter plates after 24 h of incubation. The bound dye was solubilized with ethanol, and the absorbance was measured spectrophotometrically to quantify biofilm biomass. NC: negative control.

A visible pellicle was observed on 26 of the 42 UPEC isolates (61.90%) grown in glass tubes containing artificial human urine after 24 h of incubation. These isolates exhibited a distinct adherent layer of CV–stained biomass on the inner surface of the tubes, indicating positive biofilm formation. The ODc was determined to be 1.3265. Accordingly, isolates with OD values ≤ 1.3265 were classified as non-biofilm producers, whereas those with OD values > 1.3265 were considered positive for biofilm production. A total of 26 isolates displayed OD values ≥ 1.3265, confirming biofilm production. In contrast, 16 isolates had OD values below 1.3265, indicating the absence of biofilm formation. Representative images of crystal violet–stained biofilms formed in glass tubes are presented in Figure 4. The OD values and presence or absence of biofilm are denoted in Supplementary File S8.

### 3.6. Plasmid Composition of Canine UPEC Isolate UPEC957C

**Figure 5:**
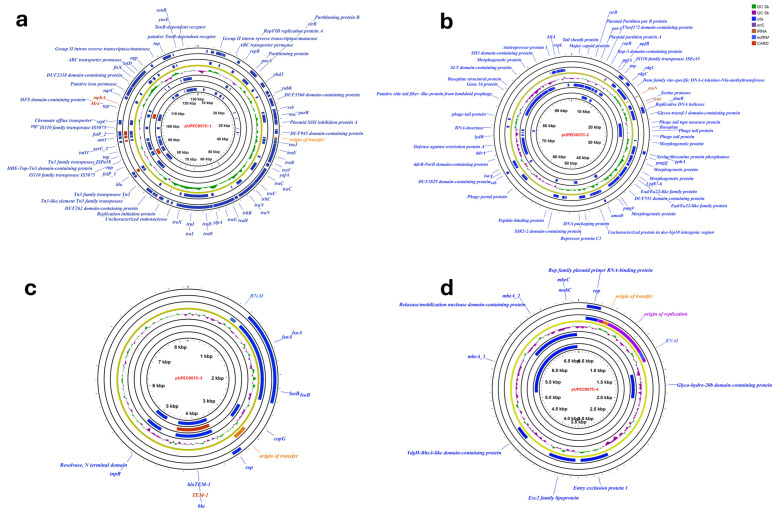
Plasmid maps of canine uropathogenic *Escherichia coli* (UPEC) strain UPEC957C (**a**) pUPEC957C-1, (**b**) pUPEC957C-2, (**c**) pUPEC957C-3, (**d**) pUPEC957C-4.

The *E. coli* isolate UPEC957C contained four plasmids (pUPEC957C-1, pUPEC957C-2, pUPEC957C-3 and pUPEC957C-4), with replicon types pO111, Col156, IncFIB(AP001918), and IncFII. The largest plasmid, pUPEC957C-1 (130,566 bp), harbored a complete *tra* gene cluster, multiple transporters including ABC permeases and chromate efflux systems, IS110- and Tn3-family transposases, and AMR genes (*mphA*, *mrx*). It also contained numerous domains of unknown function (DUF) and hypothetical proteins, indicating genomic regions whose functions had yet to be fully characterized. The second-largest plasmid (pUPEC957C-2), 99,200 bp in size, carried genes encoding morphogenetic proteins and phage-related proteins, including tail sheath protein, major capsid protein, phage tail tape measure protein, DNA packaging protein, replicative DNA helicase, portal protein, and a putative side tail fiber-like protein from lambdoid prophage-encoded genes. It also contained multiple genes encoding DUFs. The third-largest plasmid of size 8,272 bp (pUPEC957C-3) primarily harbored the AMR gene *bla*_TEM_ along with *isoA* and *isoB*. In contrast, the smallest plasmid of size 6,994 bp (pUPEC957C-4) specifically carried *mbeA_1*, *mbeA_2*, and *mbeC*. The plasmid maps are indicated in Figure 5.

### 3.7. Virulence Gene Composition of E. coli UPEC957C

The complete set of known virulence genes present in *E. coli* UPEC957C, and their corresponding functions, are summarized in Table 2. The virulence gene list identified in the *E. coli* UPEC957C is illustrated in Supplementary File S9.

### 3.8. Multilocus Sequence Typing of E. coli UPEC957C

MLST analysis of the isolate *E. coli* UPEC957C, using the Achtman 7-locus scheme via PubMLST, revealed the allelic profile *adk*36, *fumC*24, *gyrB*9, *icd*13, *mdh*17, *pur*A11, and *recA*25, corresponding to Sequence Type (ST) 73. The MLST results for *E. coli* UPEC957C are summarized in Table 3.

### 3.9. Comparative Analysis of E. coli UPEC957C Genome with Reference Strains of Human UPEC

**Figure 6:**
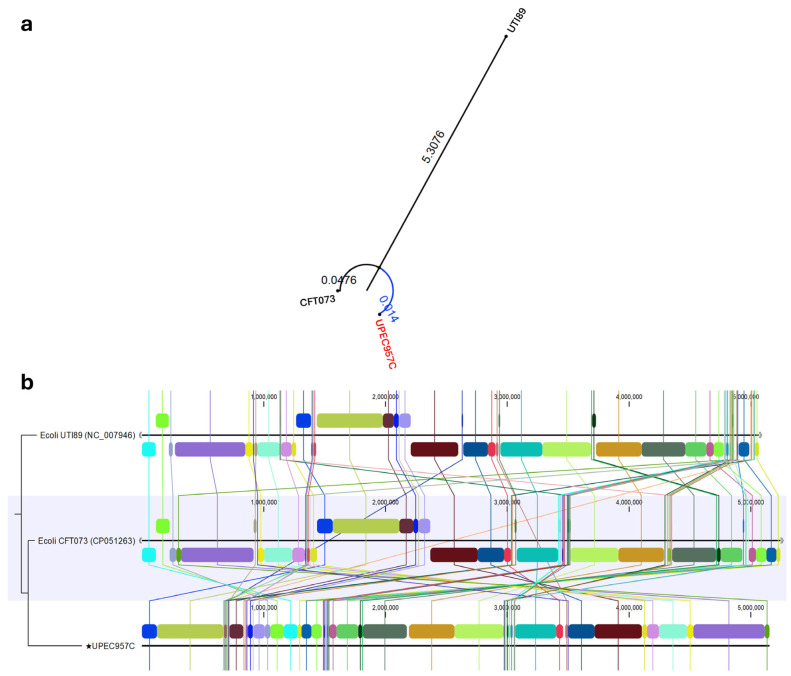
Comparative genomic analysis of UPEC957C (*) with reference uropathogenic *E. coli* (UPEC) strains. (**a**) SNP-based phylogenetic tree depicting the relationship between UPEC957C and reference strains UTI89 and CFT073. The tree was constructed using core genome single-nucleotide polymorphisms (SNPs), with branch lengths representing genetic distances. UPEC957C clusters closely with CFT073, indicating a higher genomic relatedness compared to UTI89. (**b**) Whole-genome alignment of chromosomal sequences of UPEC957C, CFT073, and UTI89. Colored blocks denote locally collinear genomic regions, and connecting lines indicate conserved synteny. The alignment shows strong collinearity between UPEC957C and CFT073, with minor genomic rearrangements relative to UTI89.

Using UPEC strain UPEC957C as the representative genome, which is of size 5,152,902 nucleotides, the SNP-based comparison with the two human UPEC strains (CFT073 and UTI89) revealed that 4,617,588 positions were shared across all three genomes, representing 89.61%. The strain CFT073 showed the highest genomic similarity to the UPEC strain UPEC957C, supported by its greater proportion of validated shared positions (97.38%). In contrast, UTI89 exhibited lower shared coverage (91.27%) compared to the UPEC strain UPEC957C, indicating that it is considerably more divergent. Overall, the SNP-based analysis demonstrates that UPEC strain UPEC957C is genetically closer to CFT073 than to UTI89, with UTI89 representing a more distantly related lineage. Figure 6 illustrates the SNP-based phylogenetic tree of the UPEC957C isolate in comparison with the reference strains UTI89 and CFT073, along with a chromosomal genome alignment of UPEC957C against these two reference strains.

The whole-genome alignment of *E. coli* strains UPEC957C, CFT073, and UTI89 further supports the SNP-based phylogenetic findings. The alignment revealed extensive collinearity and conserved genomic architecture between UPEC957C and CFT073, with only a few localized rearrangements and inversions, primarily in regions associated with mobile genetic elements and pathogenicity islands. In contrast, UTI89 exhibited several large-scale genomic rearrangements and segmental shifts, reflecting its greater evolutionary divergence from UPEC957C. The high level of sequence conservation between UPEC957C and CFT073 suggests a shared evolutionary origin and possibly similar pathogenic and host-adaptation mechanisms, whereas the structural variations observed in UTI89 may correspond to strain-specific genomic plasticity linked to niche specialization.

### 3.10. Genotypic Antimicrobial Resistance Patterns of E. coli UPEC957C

Whole-genome sequencing analysis of the UPEC isolate *E. coli* UPEC957C revealed the presence of multiple AMR genes. It harbored multiple β-lactamase genes belonging to the TEM family. Specifically, *bla_TEM-1_*, *bla_TEM-1B_*, *bla_TEM-30_*, *bla_TEM-31_*, *bla_TEM-33_*, *bla_TEM-34_*, *bla_TEM-70_*, *bla_TEM-76_*, *bla_TEM-95_*, *bla_TEM-105_*, *bla_TEM-127_*, *bla_TEM-128_*, *bla_TEM-135_*, *bla_TEM-143_*, *bla_TEM-148_*, *bla_TEM-166_*, *bla_TEM-176_*, *bla_TEM-186_*, *bla_TEM-198_*, *bla_TEM-206_*, *bla_TEM-207_*, *bla_TEM-208_*, *bla_TEM-214_*, *bla_TEM-215_*, *bla_TEM-217_*, *bla_TEM-228_*, and *bla_TEM-234_*. In addition to TEM-type β-lactamases, it carried genes conferring resistance to other antibiotic classes, including aminoglycosides (*aadA2*, *aadA3*, *aph(6)-Id*, *aph(3″)-Ib*, *aac(3)-IId*), macrolides (*mphA*), sulfonamides (*sul1*, *sul2*), trimethoprim (*dfrA12*), and tetracyclines (*tet(B)*).

Isolate UPEC957C exhibited a broad profile of efflux pump and regulatory genes, including *acrA*, *acrB*, *acrE*, *mdtE*, *emrA*, *acrD*, *yojI*, *mdtH*, *mdtG*, and *msbA*. It also carried *mrx*, a macrolide resistance gene, and *qacEdelta1* and *bacA*, which confer resistance to biocides and peptide antibiotics, respectively. In addition, this isolate harbored several regulatory genes, including *marA*, *acrS*, *emrR*, and *evgA*, which control the expression of efflux pumps and modulate bacterial stress responses. The AMR gene profile of *E. coli* UPEC957C is summarized in Table 4. Details of the AMR genes identified using CARD database and ResFinder database are provided in Supplementary File S10.

## 4. Discussion

UTIs in companion animals are frequently caused by UPEC, which possess a range of virulence factors that facilitate colonization, immune evasion, and persistence in the urinary tract. In this study, we characterized 42 clinical isolates of UPEC derived from dogs and cats, examining their AMR profiles, virulence genes, phylogenetic background, biofilm-forming ability, and plasmid composition, providing a comprehensive assessment of their urovirulence potential and zoonotic risk.

The selection of antibiotics included in the Kirby–Bauer disk diffusion panel was based on their clinical relevance and therapeutic importance in managing UTIs in companion animals, as well as their value in monitoring emerging resistance trends. Although chloramphenicol is not routinely used for the treatment of UTIs in companion animals, testing for chloramphenicol resistance provides valuable information for tracking emerging resistance trends and can indirectly indicate potential resistance to florfenicol, given their shared phenicol resistance mechanisms [34]. Our results demonstrated a substantial prevalence of phenotypic AMR among the isolates, with 40.48% resistant to at least one antibiotic and 9.52% exhibiting MDR. These findings are consistent with previous studies reporting high AMR prevalence in companion animal UPEC isolates [35]. The presence of multiple β-lactamase genes (*bla*_TEM_ variants) and efflux pump systems indicates a genomic basis for broad-spectrum resistance, highlighting the potential challenges in empirical therapy. The presence of regulatory genes controlling efflux pumps, such as *marA* and *emrR*, suggests that these isolates can dynamically respond to antibiotic pressure, enabling their persistence under such conditions and increasing their survival in clinical settings.

Phylogroup analysis revealed that the majority of isolates belonged to phylogroup B2 (69.05%), consistent with the established link between this lineage and extraintestinal pathogenicity of *E. coli* in both humans and animals [36]. B2 strains typically harbor a greater number of virulence genes, consistent with our finding that over half of the isolates carried all four urovirulence predictor genes (*vat*, *fyuA*, *chuA*, and *yfcV*). These genes contribute to iron acquisition, cytotoxicity, and adhesion, facilitating colonization and persistence within the urinary tract [12]. Similarly, the whole genome sequencing of canine isolate *E. coli* UPEC957C, belonging to B2 and a strong biofilm former, revealed the presence of fimbrial genes (*fim*, *pap*, *sfa*), curli and cellulose biosynthesis genes (*csg*, *bcs*, *wca*, *pga*), motility and flagellar genes (*fli*, *mot*, *flh*), quorum-sensing regulators (*lux*, *lsr*), stress response regulators (*soxR/S*, *oxyR/S*, *katG/E*), global transcriptional regulators (*rpo*, *csr*, *crp*, *cya*, *ihf*, *hns*), and iron acquisition systems (*iro*, *fyuA*). The co-occurrence of these genes highlights the multifactorial strategies UPEC employs to colonize and establish infection, resist host defenses, and adapt to adverse conditions, consistent with previous observations in both human and animal UPEC [7]. Biofilm formation in pooled human urine was also observed in 61.90% of isolates, confirmed by both qualitative tube-adherence and quantitative OD measurements. Biofilms are recognized as key determinants of chronicity and antibiotic tolerance in UTIs, by shielding bacteria from host defenses and antimicrobial agents. The strong biofilm-forming ability of some isolates, including canine isolate UPEC957C, likely enhances their pathogenicity and may facilitate environmental persistence, increasing the risk of transmission between animals and humans.

Plasmid analysis of canine isolate UPEC957C revealed the presence of four plasmids belonging to IncF and Col replicon types, which are well known for mediating horizontal transfer of AMR and virulence genes. The largest plasmid carries conjugation machinery, transporters, transposases, and AMR genes, facilitating horizontal gene transfer and dissemination of resistance traits. The second-largest plasmid-encoded phage-related and morphogenetic proteins reflect prophage-derived contributions to genome plasticity, while smaller plasmids harbor AMR genes and pili-associated genes (*mbeA*, *mbeC*), enhancing survival, adherence, and persistence. These findings pose a potential public health concern, given the close human-pet animal interface in household environments [5]. Importantly, comparative genomic analysis between our sequenced canine UPEC strain *E. coli* UPEC957C and two human UPEC strains demonstrated that UPEC957C is closely related to human UPEC genomes, sharing 97.38% nucleotides with CFT073, a strain isolated from a human patient with acute pyelonephritis [37] and 91.27% with UTI89, a strain isolated from a human patient with cystitis [38]. MLST confirmed that UPEC957C belongs to Sequence Type 73 (ST73), a lineage commonly implicated in human UTIs.

Overall, this study demonstrated that companion animal UPEC isolates are characterized by a combination of AMR and virulence traits, and biofilm-forming ability in human urine, with a predominance of B2 phylogroup strains harboring multiple plasmids and sharing substantial genomic similarity with human UPEC strains. These findings underscore the importance of antimicrobial stewardship, routine surveillance, and effective biosecurity measures in mitigating the risk of transmission of highly virulent and resistant UPEC strains between animals and humans. Furthermore, our results emphasize the need for targeted therapeutic approaches that account for both resistance profiles and virulence potential, particularly in isolates exhibiting strong biofilm formation.

## 5. Conclusions

UPEC isolates originating from companion animals exhibit high pathogenic and zoonotic potential, with predominance of phylogroup B2 strains, multiple virulence genes, strong biofilm formation in human urine, and diverse plasmid-mediated AMR. These traits enable adherence, colonization, and persistence in the urinary tract, contributing to chronic and recurrent infections. The coexistence of virulence and MDR determinants raises significant therapeutic and epidemiological concerns, as it may facilitate the horizontal transfer of these genes to other bacteria. Importantly, the zoonotic and reverse zoonotic potential of these strains poses a significant public health risk to pet owners due to close contact between pets and humans. The findings of this study suggest a potential zoonotic risk; however, this remains to be confirmed through further studies. Further, these findings highlight the importance of responsible antimicrobial use, routine molecular surveillance of UPEC, enhanced hygiene measures, and integrated One Health–oriented strategies to mitigate the risk of zoonotic transmission and preserve the efficacy of antimicrobial therapies in both veterinary and human medicine.

## Figures and Tables

**Table 1:** Patient demographics and year of UPEC isolation.

Category	Canine (*n* = 34)	Feline (*n* = 8)	Total (*n* = 42)
**Sex**			
Male	8	6	14
Female	26	2	28
**Age Group**			
<1 year	9	0	9
1–5 years	3	2	5
6–10 years	12	2	14
>10 years	9	3	12
N/A	1	1	2
**Year of Isolation**			
2023	9	2	11
2024	25	6	31

N/A: Not available; indicates cases where age information was not recorded or could not be determined from available records.

**Table 2:** Virulence genes identified in *E. coli* UPEC957C and their associated functions.

Gene	Function	Reference
*fim A*, *B*, *C*, *D*, *E*, *F*, *G*, *H*, *I*	Mediates assembly and function of type 1 fimbriae, enabling adhesion to uroepithelial cells	[16]
*pap A*, *B*, *C*, *D*, *F*, *G-II*, *H*, *I*, *J*, *K*, *X*	Mediates assembly and function of P fimbriae, enabling adhesion to kidney epithelial cells	[17]
*sfa D*, *Y*, *X*	Mediates assembly and function of S fimbriae, enabling adhesion to urinary tract and endothelial epithelial cells	[18]
*csg A*, *B*, *C*, *D*, *E*, *F*, *G*	Mediates curli fimbriae production, promoting adhesion to surfaces and biofilm formation	[19]
*bcs A*, *B*, *C*, *E*, *F*, *G*, *Q*, *R*, *Z*	Mediates cellulose biosynthesis, contributing to biofilm formation and surface adhesion	[20]
*wca A*, *B*, *C*, *D*, *E*, *F*, *I*, *J*, *K*, *L*, *M*	Mediates colanic acid biosynthesis, contributing to biofilm formation and protection against environmental stress	[21]
*pga A*, *B*, *C*, *D*	Mediates synthesis and export of poly-β-1,6-N-acetyl-D-glucosamine (PGA), promoting biofilm formation and surface adhesion	[22]
*ycg B*, *J*, *L*, *M*, *N*, *R*, *X*, *Z*	Biofilm regulators influencing matrix production under acidic stress	[23]
*ymg C*, *D*, *E*, *G*	Regulates biofilm formation and curli expression	[23]
*fli A*, *D*, *E*, *F*, *G*, *H*, *I*, *J*, *K*, *L*, *M*, *N*, *O*, *P*, *Q*, *R*, *S*, *T*, *Z*	Mediates flagellar assembly and motility	[24]
*mot A*, *B*	Generate flagellar rotation for bacterial movement	[25]
*flh A*, *B*, *C*, *D*, *E*	Regulates flagellar assembly and controls expression of flagellar genes	[24]
*lux R*, *S*	Mediate quorum sensing, regulating biofilm formation, and virulence	[16]
*lsr K*, *N*, *C*	Mediate quorum sensing, facilitating biofilm formation and regulation of virulence genes	[26]
*rpo A*, *B*, *C*, *D*, *E*, *H*, *N*, *S*	Encode RNA polymerase subunits and sigma factors, regulating transcription of genes involved in growth, stress response, and virulence	[27]
*csr B*, *C*, *D*	Regulate the csrA global regulatory system, controlling biofilm formation, motility, and virulence gene expression	[28]
*crp*	Transcriptional regulator controlling carbon metabolism, virulence, and biofilm formation	[29]
*cya A*, *R*, *Y*	Modulate cAMP levels and regulatory pathways, controlling metabolism, virulence, and biofilm formation	[30]
*ihf A*, *B*	Binds DNA to regulate transcription, virulence gene expression, and biofilm formation	[16]
*hns*	Global transcriptional repressor modulating virulence genes, biofilm formation, and stress response	[16]
*iro B*, *C*, *D*, *E*, *N*	Mediate salmochelin siderophore biosynthesis, transport, and uptake, promoting iron acquisition and virulence.	[31]
*fyuA*	Functions as an outer membrane receptor for yersiniabactin, mediating iron uptake	[32]
*sox R*, *S*	Regulate oxidative stress response and activate genes involved in detoxification, survival, and virulence	[33]
*oxy R*, *S*	Regulate oxidative stress response and activate genes for detoxification, survival, and virulence	[33]
*kat G*, *E*	Detoxify hydrogen peroxide and protect against oxidative stress, enhancing survival and virulence in the urinary tract	[33]

**Table 3:** MLST allelic profile of the *Escherichia coli* UPEC957C UPEC isolate using the Achtman scheme.

Locus	Allele	Length	Start Position	End Position
*adk*	36	536	48,63,474	4,864,009
*fumC*	24	469	412,721	413,189
*gyrB*	9	460	2,135,810	2,136,269
*icd*	13	518	4,135,147	4,135,664
*mdh*	17	452	2,660,853	2,661,304
*purA*	11	478	1,556,140	1,556,617
*recA*	25	510	3,363,085	3,363,594

**Table 4:** Genomic distribution of AMR determinants in *Escherichia coli* UPEC957C.

AMR Category	AMR Gene/s
β-lactam resistance	*bla_TEM-1_*, *bla_TEM-1B_*, *bla_TEM-30_*, *bla_TEM-31_*, *bla_TEM-33_*, *bla_TEM-34_*, *bla_TEM-70_*, *bla_TEM-76_*, *bla_TEM-95_*, *bla_TEM-105_*, *bla_TEM-127_*, *bla_TEM-128_*, *bla_TEM-135_*, *bla_TEM-143_*, *bla_TEM-148_*, *bla_TEM-166_*, *bla_TEM-176_*, *bla_TEM-186_*, *bla_TEM-198_*, *bla_TEM-206_*, *bla_TEM-207_*, *bla_TEM-208_*, *bla_TEM-214_*, *bla_TEM-215_*, *bla_TEM-217_*, *bla_TEM-228_*, *bla_TEM-234_*
Aminoglycoside resistance	*aadA3*, *aadA2*, *aph(6)-Id*, *aph(3″)-Ib*
Macrolide resistance	*mphA*
Sulfonamide resistance	*sul1*, *sul2*
Trimethoprim resistance	*dfrA12*
Efflux pumps	*acrA*, *acrB*, *acrE*, *mdtE*, *emrA*, *acrD*, *yojI*, *mdtH*, *mdtG*, *msbA*
Biocide/other resistance	*mrx*, *qacEdelta1*, *bacA*
Regulators/transcription factors	*marA*, *hns*, *leuO*, *acrS*, *emrR*, *evgA*, *crp*, *gadW*, *cpxA*

## Data Availability

The original contributions presented in this study are included in the article/Supplementary Material. Further inquiries can be directed to the corresponding author.

## Supplementary Materials

The following supporting information can be downloaded at: https://www.mdpi.com/article/10.3390/tropicalmed11040101/s1, File S1: Primer Sequences Used for PCR Amplification of Urovirulence Genes and Phylo-typing; File S2: Detailed Demography of Host Animals Collected from the Clinical Microbiology, Parasitology, and Serology Laboratory, UF-VH; File S3: Antimicrobial Susceptibility Test Results of UPEC Isolates; File S4: Urovirulence Genes Distribution in 42 UPEC Isolates; File S5: Gel Images of Urovirulence Gene Amplifications; File S6: Distribution of Phylogenetic Markers and Subsequent Phylogroups of UPEC isolates; File S7: Gel Images of Phylogenetic Marker Amplifications; File S8: The Optical Density Values and Biofilm Production Data; File S9: Virulence Gene profiles of UPEC957C Isolate Identified Using Virulence Factor Database (VFDB); File S10: Details of the AMR Genes Identified Using CARD and ResFinder Database.

## Abbreviations

The following abbreviations are used in this manuscript:

UPEC	Uropathogenic *Escherichia coli*
UTI	Urinary tract infection
AMR	Antimicrobial resistance
AST	Antimicrobial susceptibility testing
HGT	Horizontal gene transfer
MDR	Multidrug resistance
UF-VH	University of Florida-Veterinary Hospitals
FOX	Cefoxitin
FEP	Cefepime
TE	Tetracycline
D	Doxycycline
GM	Gentamicin
AN	Amikacin
IPM	Imipenem
C	Chloramphenicol
PCR	Polymerase chain reaction
PBS	Phosphate-buffered saline
CV	Crystal violet
OD	Optical density
ODc	Optical density cut-off
SD	Standard deviation
ONT	Oxford nanopore technology
UDI	Unique dual indices
CARD	Comprehensive antibiotic resistance database
VFDB	Virulence factor database
MLST	Multilocus sequence typing
SNP	Single nucleotide polymorphism
