# Supplementary figures and images for: Characterization of Antimicrobial Resistance and Potential Zoonotic Risk in Uropathogenic Escherichia coli Isolated from Companion Animals, with Genomic Analysis of Virulence Determinants in a Representative Isolate
Source: Trop Med Infect Dis. 2026 Apr 13;11(4):101. doi: 10.3390/tropicalmed11040101 (PMC13120572; doi:10.3390/tropicalmed11040101)

Gel Images of Urovirulence Gene Amplifications

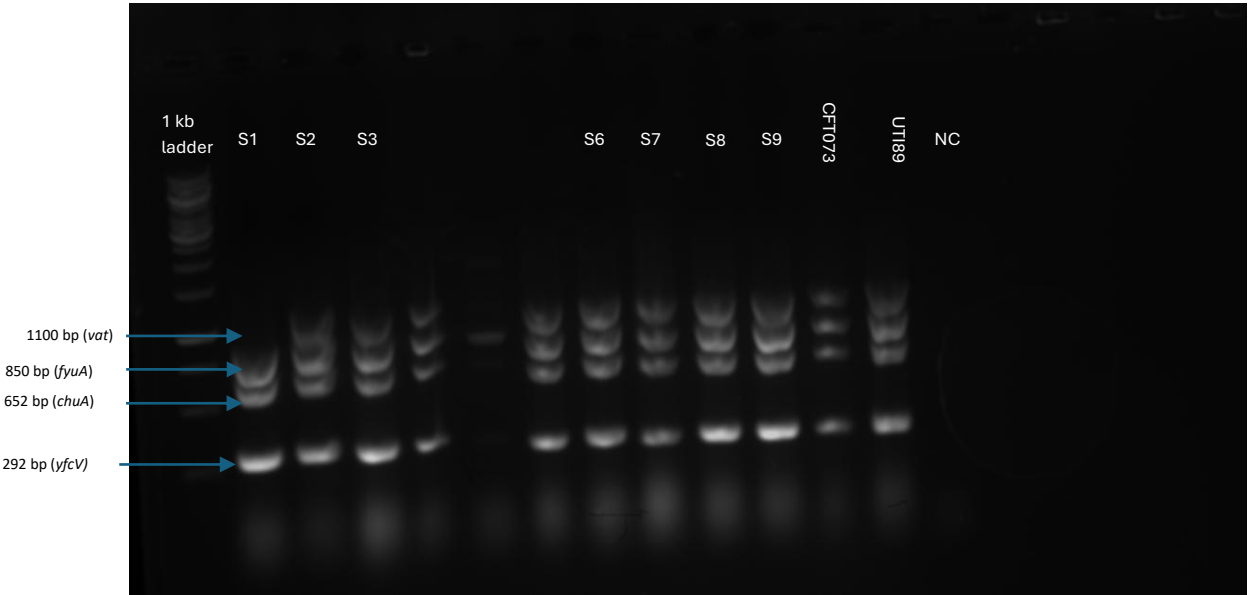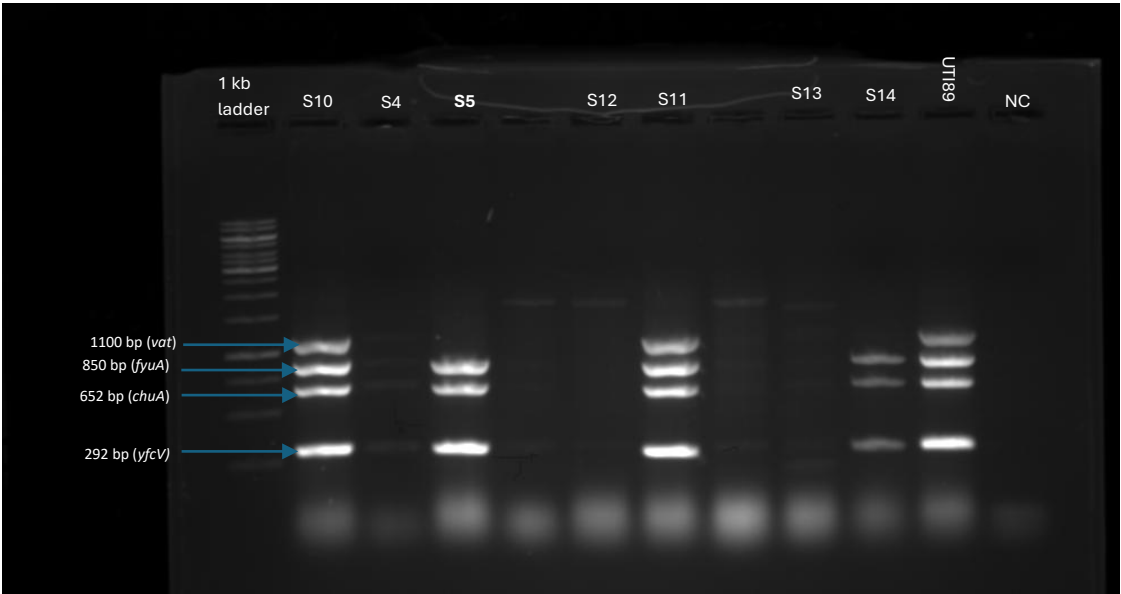

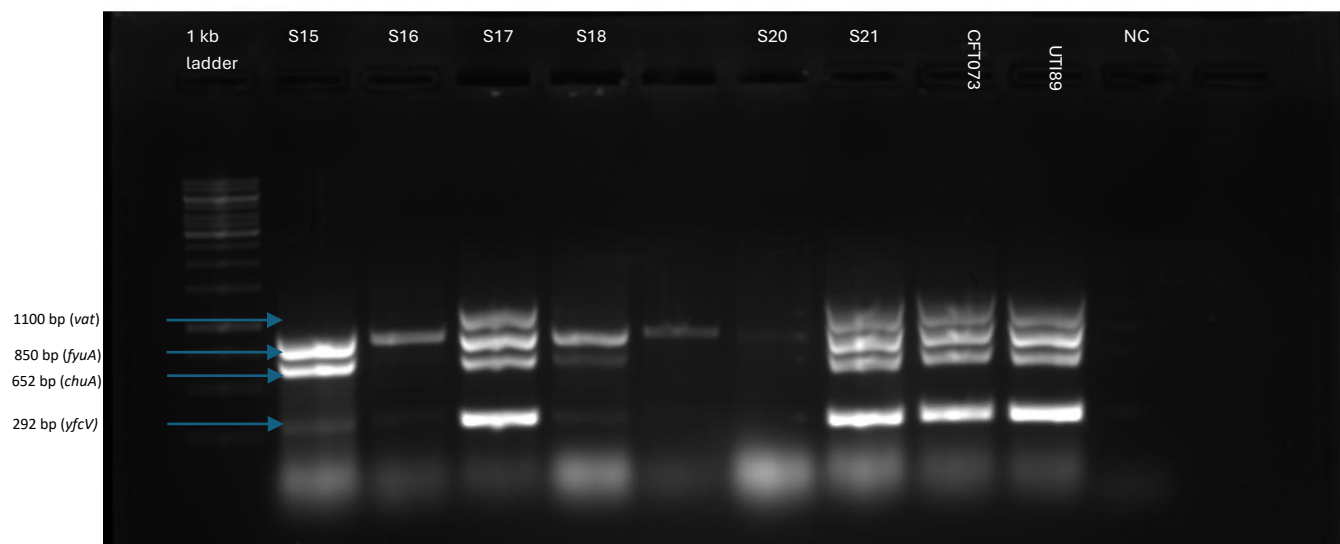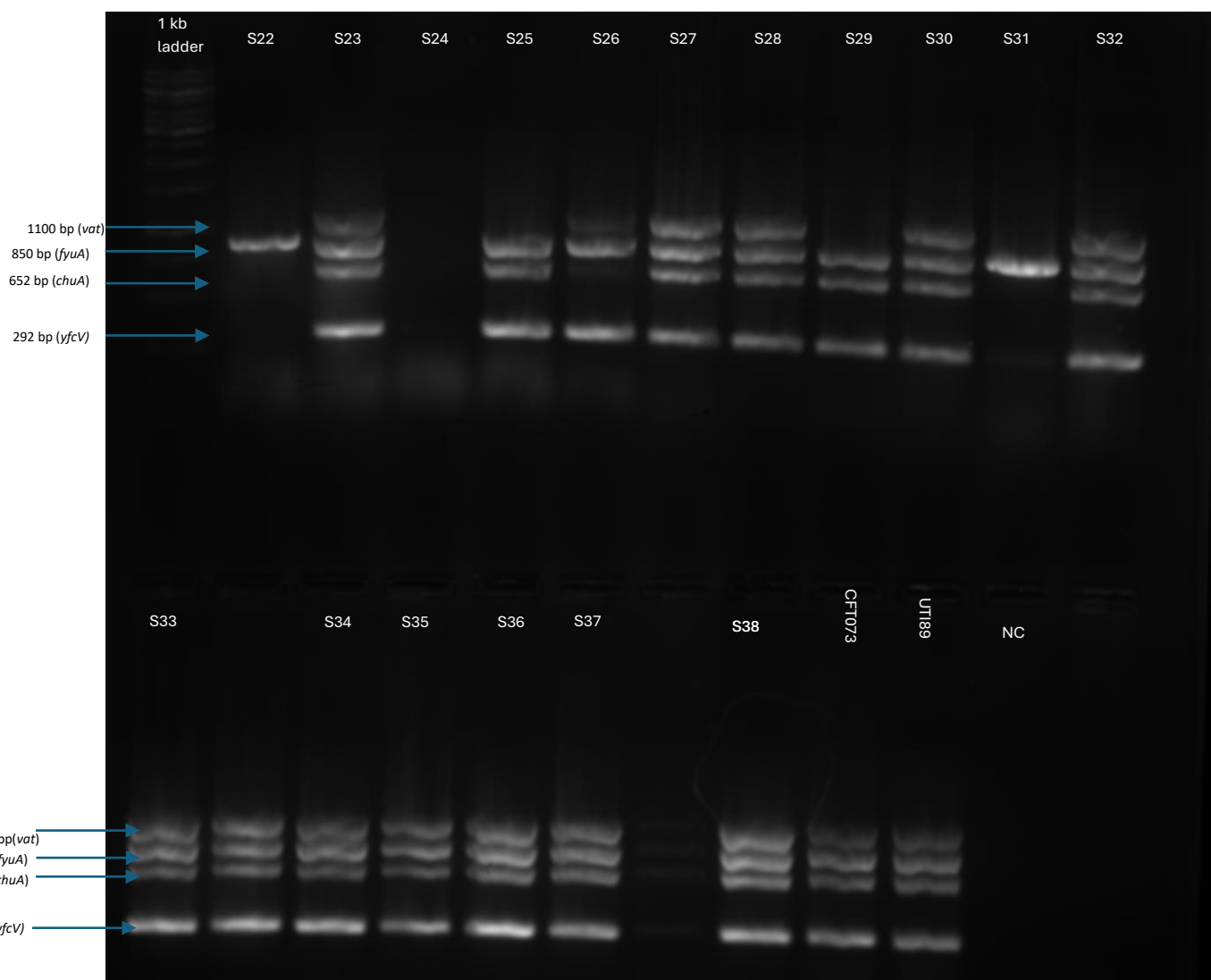

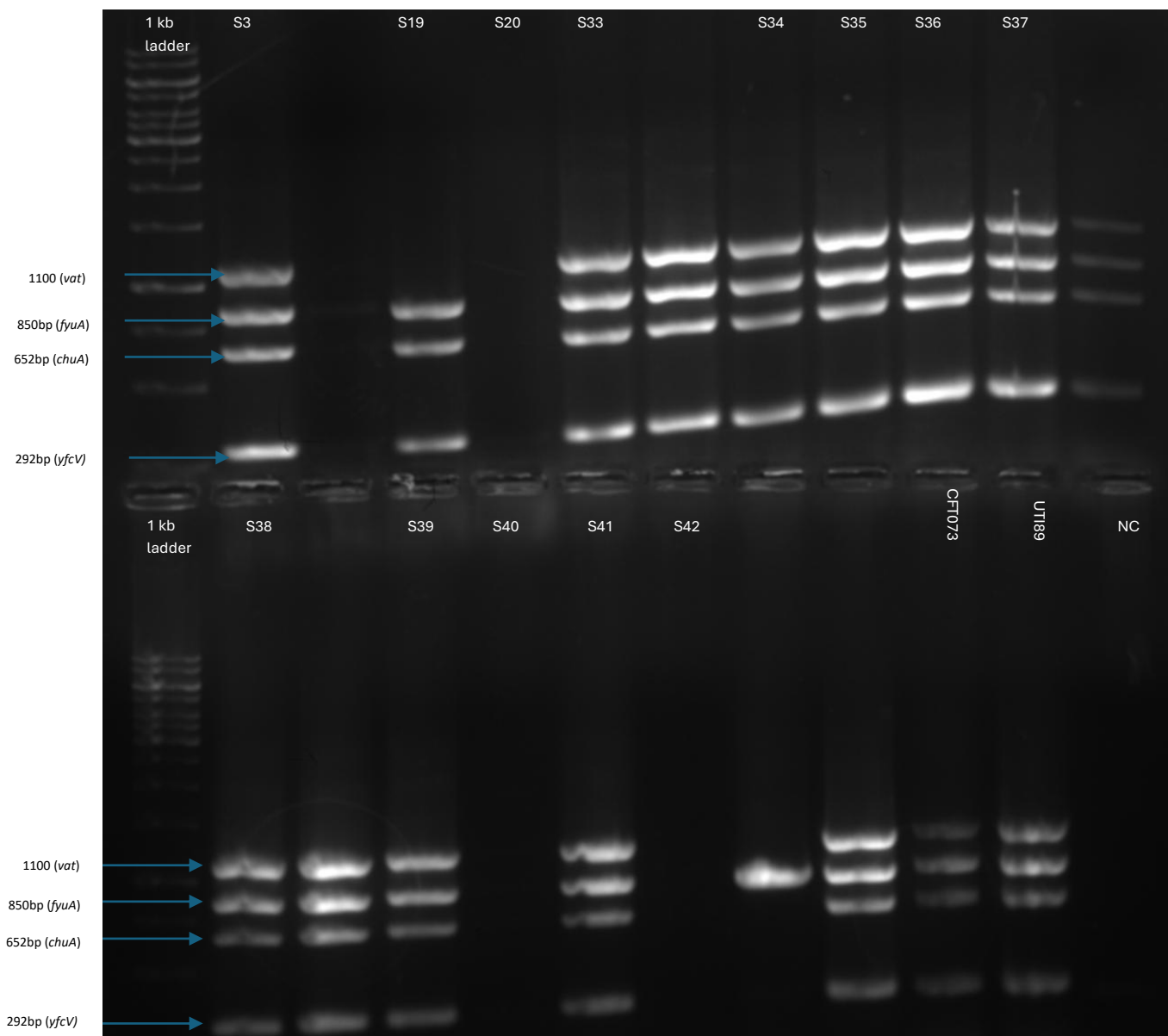

Supplement: Supplementary file 1 [file tropicalmed-11-00101-s001.zip › Supplementary Files_UPEC/Supplementary File S5.pdf]

Gel Images of Phylogenetic Marker Amplifications

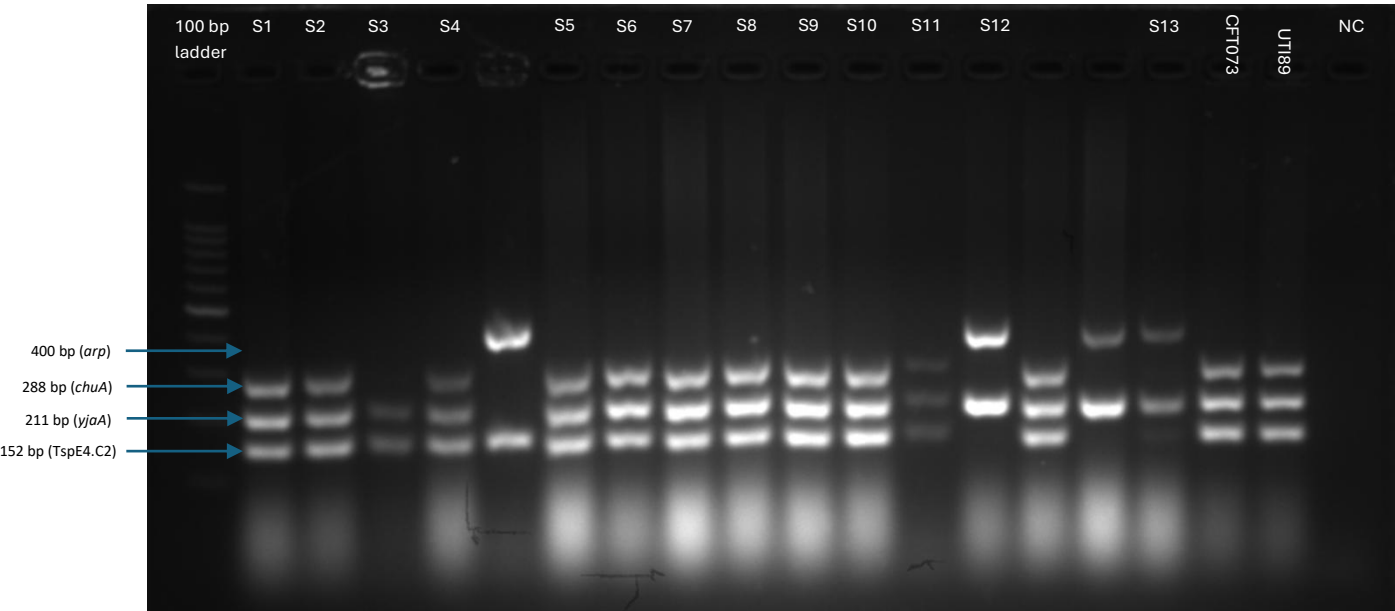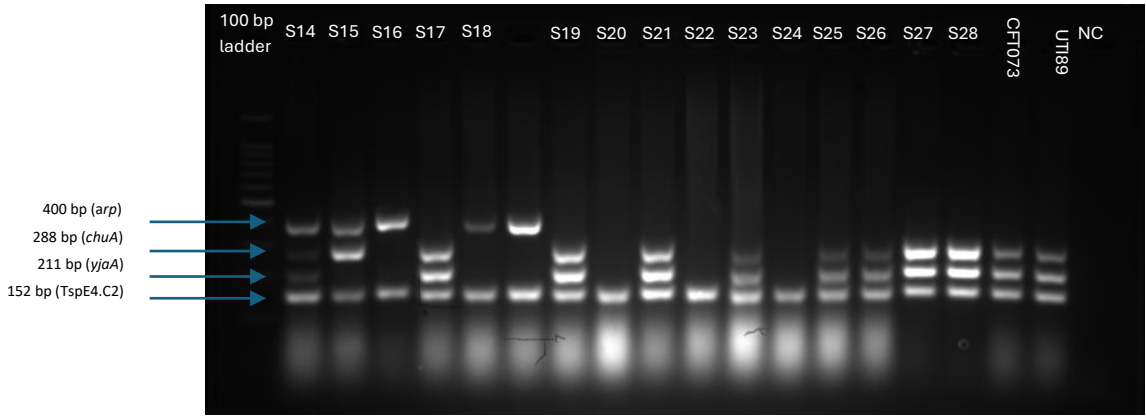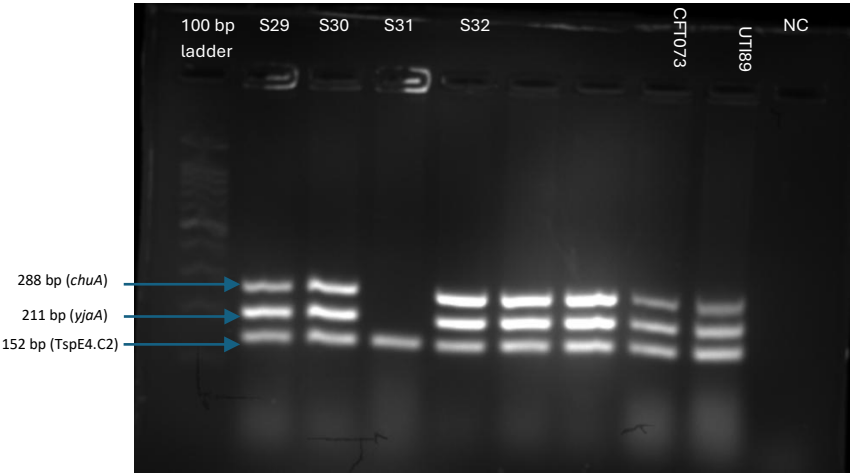

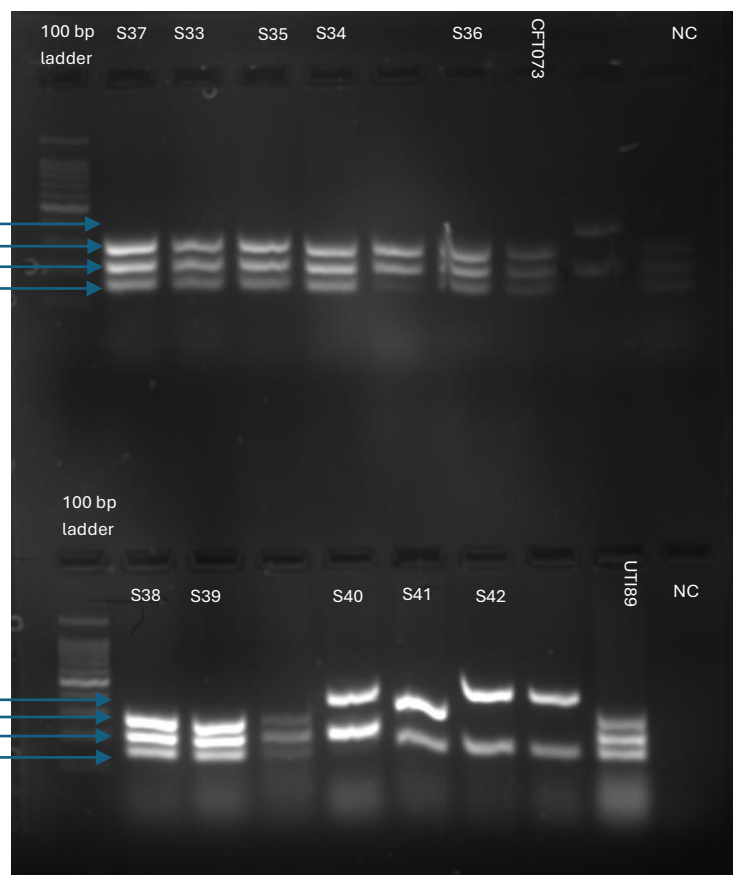

Supplement: Supplementary file 1 [file tropicalmed-11-00101-s001.zip › Supplementary Files_UPEC/Supplementary File S7.pdf]
